# Impairment of Retrograde Neuronal Transport in Oxaliplatin-Induced Neuropathy Demonstrated by Molecular Imaging

**DOI:** 10.1371/journal.pone.0045776

**Published:** 2012-09-20

**Authors:** Dawid Schellingerhout, Lucia G. LeRoux, Brian P. Hobbs, Sebastian Bredow

**Affiliations:** 1 Departments of Radiology and Experimental Diagnostic Imaging, The University of Texas MD Anderson Cancer Center, Houston, Texas, United States of America; 2 Department of Experimental Diagnostic Imaging, The University of Texas MD Anderson Cancer Center, Houston, Texas, United States of America; 3 Department of Biostatistics, The University of Texas MD Anderson Cancer Center, Houston, Texas, United States of America; University of Cambridge, United Kingdom

## Abstract

**Background and Purpose:**

The purpose of our study was to utilize a molecular imaging technology based on the retrograde axonal transport mechanism (neurography), to determine if oxaliplatin-induced neurotoxicity affects retrograde axonal transport in an animal model.

**Materials and Methods:**

Mice (n = 8/group) were injected with a cumulative dose of 30 mg/kg oxaliplatin (sufficient to induce neurotoxicity) or dextrose control injections. Intramuscular injections of Tetanus Toxin C-fragment (TTc) labeled with Alexa 790 fluorescent dye were done (15 ug/20 uL) in the left calf muscles, and *in vivo* fluorescent imaging performed (0–60 min) at baseline, and then weekly for 5 weeks, followed by 2-weekly imaging out to 9 weeks. Tissues were harvested for immunohistochemical analysis.

**Results:**

With sham treatment, TTc transport causes fluorescent signal intensity over the thoracic spine to increase from 0 to 60 minutes after injection. On average, fluorescence signal increased 722%+/−117% (Mean+/−SD) from 0 to 60 minutes. Oxaliplatin treated animals had comparable transport at baseline (787%+/−140%), but transport rapidly decreased through the course of the study, falling to 363%+/−88%, 269%+/−96%, 191%+/−58%, 121%+/−39%, 75%+/−21% with each successive week and stabilizing around 57% (+/−15%) at 7 weeks. Statistically significant divergence occurred at approximately 3 weeks (p≤0.05, linear mixed-effects regression model). Quantitative immuno-fluorescence histology with a constant cutoff threshold showed reduced TTc in the spinal cord at 7 weeks for treated animals versus controls (5.2 Arbitrary Units +/−0.52 vs 7.1 AU +/−1.38, p<0.0004, T-test). There was no significant difference in neural cell mass between the two groups as shown with NeuN staining (10.2+/−1.21 vs 10.5 AU +/−1.53, p>0.56, T-test).

**Conclusion:**

We show–for the first time to our knowledge–that neurographic *in vivo* molecular imaging can demonstrate imaging changes in a model of oxaliplatin-induced neuropathy. Impaired retrograde neural transport is suggested to be an important part of the pathophysiology of oxaliplatin-induced neuropathy.

## Introduction

Oxaliplatin is a platinum-based chemotherapeutic that is widely used in the treatment of colon carcinoma [1]. Its principal and dose-limiting side-effect is neurotoxicity, which occurs as an acute transient post-infusion syndrome, or as a chronic cumulative sensory neuropathy [2], [3]. The clinical presentation is of a glove-and-stocking distribution sensory loss with paresthesias, dysesthesias and pain, particularly pain induced by exposure to cold [4]. The majority of patients treated with oxaliplatin will experience some degree of neuropathy, with allopathy induced by cold and touch being the leading symptoms. Patients frequently describe this neuropathy as: “walking on knives”, and it is a major cause of cancer treatment-related morbidity.

Current modalities of investigation in oxaliplatin-induced neuropathy are the clinical neurologic exam, supplemented by electrophysiologic studies, both of which become abnormal in the majority of patients on oxaliplatin therapy (reviewed in [5]). The weaknesses of these current methodologies are the relatively subjective nature of the neurologic exam (there are few objective neurologic signs in these patients who have predominantly sensory symptoms), and the relatively late appearance of objective electrophysiological signs. The most important weakness though, is the inability of current investigative methods to distinguish reliably between the various pathophysiological mechanisms of nerve injury.

The mechanism by which oxaliplatin induces neuropathy is currently unknown, but there are several leading hypotheses:

- The channelopathy hypothesis poses that oxaliplatin leads to ion channel dysfunction and hence to nerve dysfunction and clinical neuropathy [4], [6]. In keeping with this hypothesis, calcium/magnesium infusions have been proposed to prevent oxaliplatin neurotoxicity, but with controversy as to its effects on the effectiveness of chemotherapy (reviewed in [2]). Anti-convulsants and other channel acting drugs have also been proposed and tested (reviewed in [7]). Mexiletine, an orally available sodium channel blocker has been used in animal studies to relieve mechanical allodynia and cold hyperalgesia due to oxaliplatin neuropathy [8].
- The growth factor deficiency hypothesis is supported by observations that externally administered growth factors appears to protect against oxaliplatin neurotoxicity in animal cell culture experiments [9]. An experimental agent, BNP7787, currently in clinical trials, can apparently protect against platinum-group neuropathy, and does this by -among other mechanisms- stabilizing microtubules thought to be important in growth factor physiology [10]. Neurotropin, a non-protein extract from inflamed rabbit skin, alleviated some aspects of oxaliplatin neuropathy in animal and cell studies [11].
- The inflammatory hypothesis of neuropathy holds that an inappropriate inflammatory response to chemotherapy and cancer damages nerves. Vitamin E, a known anti-oxidant thought to be protective against free radical induced inflammatory damage agent, has been tested in a double blind clinical trial for chemoprevention of neuropathy by a variety of neurotoxic agents, but was found to be ineffective [12].
- Mitochondrial toxicity was recently proposed as a mechanism to explain selective neurotoxicity in sensory nerves [13], [14].

Thanks to behavioral and biochemical studies done by prior investigators [15], [16], [17], [18], [19], we have excellent animal models of platinum group chemotherapy agent neurotoxicity, with detailed published chemotherapy regimens and histological as well as behavioristic correlations. However, none of these models have previously been studied by means of imaging, because a suitable imaging technology was lacking.

There is a great **need** for an investigative technology that is: a) informative of the basic pathophysiological mechanisms of nerve injury, and b) allows repeated non-invasive assessments allowing the outcomes of interventions to be measured, c) early in the process of nerve injury, while outcomes could perhaps still be altered.

In this paper we address this need by demonstrating the use of a molecular imaging agent based on the retrograde axonal transport mechanism in the setting of oxaliplatin-induced neuropathy. We show that a molecular imaging tracer: a) can demonstrate retrograde neuronal transport impairment in oxaliplatin toxicity, b) using a non-invasive imaging measurement, that can be repeated, allowing longitudinal studies, and c) that imaging changes occur relatively early in the course of the development of neuropathy. Our work suggests impaired retrograde transport to be an important part of the pathophysiology of oxaliplatin-induced neuropathy.

## Methods

### Animals and Chemotherapy Regimen

This study was approved by our institutional animal care review board. We used two groups of 8 BALB/C mice (Charles River Laboratories, Wilmington, MA) for imaging studies: controls and oxaliplatin treatment (16 animals total for imaging studies). An additional two groups of 4 animals each were used for histological studies (8 animals total for histology), euthanized 7 weeks after starting the oxaliplatin regimen. Using a well-characterized model system described in the literature [20], experimental animals received Oxaliplatin (Sigma-Aldrich, St Lois, MO) dissolved in sterile water at 1 mg/ml (diluted as needed in 5% glucose) at a dose of 3 mg/kg per intra-peritoneal injection (0.2 ml total) for 5 days during the first week, followed by a 7 day rest, followed by another series of 5 daily injections in the third week. The total dose was 30 mg/kg cumulative, spread out over 10 injections given in two cycles. Control animals received 5% glucose sham injections. Animal weights were determined once a week at the end of the week prior to injections.

### Imaging Agent

Recombinant TTc was isolated from *E. coli* carrying the expression plasmid pAE-Fc (a kind gift of Dr. Ana L. T. O. Nascimento [21]) and is identical to the original published sequences by Fairweather and co-workers [22], [23]. The 50-kDa His-tagged protein was purified in its native state using standard procedures [24], [25] and labeled with Alexa Fluor® succinimidyl esters 790 according to the manufacturer’s protocol (Invitrogen; Carlsbad, CA).

### In vivo Imaging

**Figure 1:**
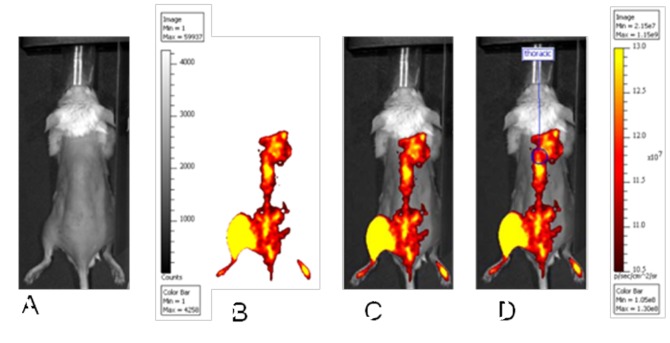
Fluorescent Imaging Methodology. A grayscale white light image captured while the system’s illumination lights are on (A) while the pseudocolor image of fluorescent photon flux in the near-infrared range showing the location of TTc-related fluorescence (B). The images shown in A and B can be combined in an overlay image (C). The Region-of-Interest (ROI) measurements can be taken to quantify photon flux (D). In this example, images are from an animal 60 min after TTc-injection into the calf muscles.

In vivo imaging was done at baseline and weekly thereafter for 5 weeks, with 2-week intervals between the final time points at 7 and 9 weeks: All in vivo imaging was done with the Xenogen IVIS 200 fluorescent small animal imager (Caliper Life Sciences, Hopkinton, MA) using bandpass excitation and emission filters for near infrared light (ICG set for Alexa Fluor® 790, 710–760/810–875 nm) (Fig. 1). Animals were anesthetized using isoflurane (Abbott Laboratories, North Chicago, IL), and their dorsal fur was removed with hair removal cream. The mice underwent injections in their left calf muscles with 15 μg (in 20 μl physiological dextrose solution) of TTc-790 using Hamilton glass syringes with 26G needles mounted on a stereotactic frame. Images of the animals’ dorsal aspects were taken every 10 minutes at 30, 40, 50, and 60 minutes post TTc injection (0.5 sec/f = 4 exposure with binning level set at medium, and field of view of 13.1 cm for fluorescence; 0.2 sec/f = 8 exposure with identical field of view for white light photographs). Images were analyzed using Living Image 3.2 systems software (Caliper Life Sciences). Region of Interest (ROI) measurements were done over the lower thoracic spine at baseline and through 60 minutes after injection, with photon flux measured in photons per second per centimeter square per steradian (p s^−1^ cm^−2^ sr^−1^) (see Fig. 1).

### Histology

Animals were killed for histology at 7 weeks after the start of oxaliplatin treatment. Both control (n = 3) and oxaliplatin (n = 3) treated animals were euthanized 1 hour after the injection of TTc in the calf muscles and the spines with cords were excised and frozen on dried ice. We embedded 1 cm pieces of the complete spinal column ranging from the lower thoracic cord (T12–T13), the complete lumbar area (L1-L6) as well as the higher sacral (S1–S3) area for cryotomy. Serial frozen sections were cut at a thickness of 30 μm using a cryostat. For immunohistochemical detection of TTc and neuron – specific nuclear protein (NeuN), the cryosections were fixed with 4% paraformaldehyde for 5 minutes, sections were blocked in a solution of phosphate-buffered saline (PBS), 1% bovine serum albumin, and 0.03% Triton X-100. The primary detection was done with a polyclonal rabbit anti-TTc antibody (1 μg/ml; Rockland Immunochemicals, Gilbertsville, PA) in PBS for 1 hour at room temperature while the secondary immuno reaction included a simultaneous reaction of anti-NeuN (MAB377X, Millipore, Temucila, CA at 1∶100) and a secondary goat anti-rabbit Alexa Fluor 546–labeled antibody (1 μg/ml, Invitrogen) at room temperature for 1hour. For detection of fluorescence, sections were mounted in Prolong Gold anti-fade reagent with DAPI (4′,6-diamidino-2-phenylindole, a fluorescent stain that binds strongly to DNA). Micrographs were imaged under identical conditions using a10x UPLAPO objective with an inverted Olympus FV1000 scanning confocal microscope (Olympus America Inc, Center Valley, PA). In this study, we used a FV5-LD405 laser diode (Olympus) and both the argon ion 488 nm and green HeNe 543.5 nm lasers (Melles Griot,Albuquerque, NM) and suitable filter sets (DM405/488/543 nm).

### Immunohistochemistry Analysis

We studied 20 cryo-sections per animal and used the Spinal Cord Atlas [26] for histological orientation. The effect of imaging artifacts on analysis was minimized by selecting 3 near perfect immuno-histochemistry sections per immuno-stain (anti-NeuN and anti-TTc) for both treated and untreated animals. All digital imaging manipulations were done using ImageJ (rsbweb.nih.gov). Unmodified RGB bitmap images were converted to 16 bit grayscale images, and background fluorescent signal was removed by means of a thresholding procedure that removed all signal below a user-selected cutoff. The cutoff value was selected visually on a training set of images until a value was found that captured positive staining but excluded background signal. For NeuN a cutoff of 22, and for TTc a cutoff of 15 was found to adequately filter out background, and these values were used consistently for all our quantitative analysis. The mean fluorescence intensity in arbitrary units (AU) values (as provided by ImageJ) for each histology section were recorded and analyzed using a spreadsheet.

### Statistics

Student’s paired T-test was done to assess the statistical significance of animal weights, fluorescent histology pixel counts and for simple comparisons of fluorescent values using a spreadsheet (Excel, Microsoft, Redmond, WA). Fluorescent uptake curves over the thoracolumbar spine for oxaliplatin-treated and control animals were evaluated formally by analyzing the observed change from 0 minutes to 60 minutes post TTc injection in fluorescent signal intensity using a linear mixed-effects regression model. The statistical software R (R Development Core Team, http://www.r-project.org) version 2.12.0 with package “nlme” was used for statistical analysis. The model accounts for correlation among within animal measurements over time, as well as the quadratic trend over time in mean TTc uptake as a function of week.

## Results

### Animals

No mortality was observed over the course of the study. Oxaliplatin-treated animals demonstrated significant weight loss to a maximum of 1.48 g (p<0.05, T-test) compared to 0.66 g for controls (from baselines of 22.57 g and 23.49 g). By the end of week 5, weights of most animals had again reached pre-injection values. No other phenotypic changes were observed in the animals.

### Imaging Agent

The properties of labeled TTc were compared to unaltered TTc (Roche Applied Science; Indianapolis, IN) by Western blot and cell uptake studies using PC-12 pheochromocytoma cells (ATCC; Manassas, VA) and found to be identical, as shown in prior work by our group [27].

### In vivo Imaging

Using the methodology demonstrated in Fig. 1, fluorescently labeled imaging agent was observed migrating from the injection site in the calf muscle to the spinal cord.

**Figure 2:**
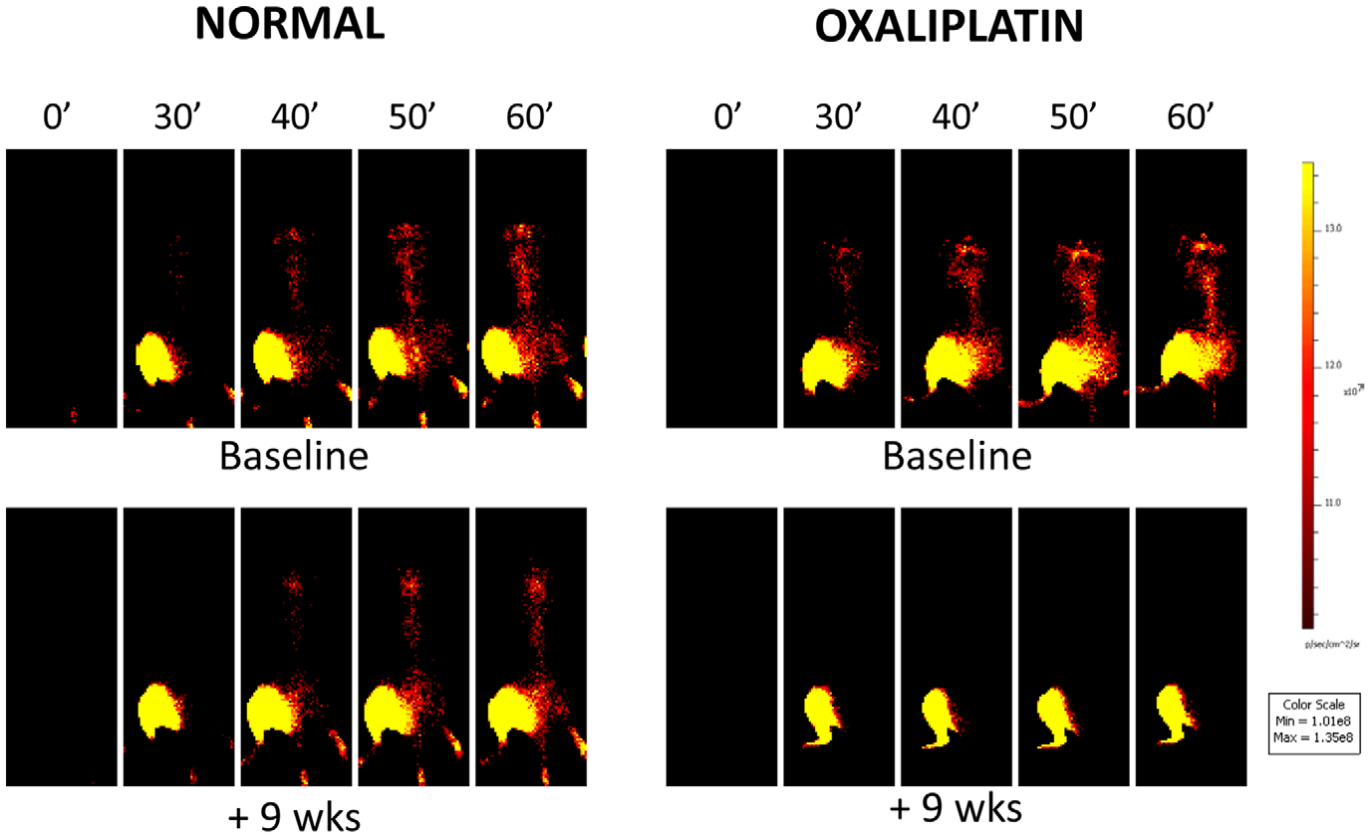
TTc transport under normal and chemotoxic conditions as demonstrated by imaging, shows impairment in Oxaliplatin-treated animals. Representative fluorescent images shows the time course of TTc uptake after left calf injection in normal (left panels) and oxaliplatin treated animals (right panels). At baseline, the uptake is identical (top panels) with migration of TTc occurring from the injection site to the spinal cord over 60 minutes. TTc fluorescent signal is always visible over the injection site in the calf, but there are significant variations in the subsequent transport of TTc between controls and treated animals. Sham treated animals maintain cord uptake similar to that seen at baseline (note the stippled ovals in the left panels at 60 minutes). During the course of oxaliplatin treatment, however, there is a progressive decrease in the amount of transport of TTc to the spinal cord (note the red stippled ovals in the right set of panels at 60 minutes). ROI measurements over the thoracic spine will yield a time-signal curve that can be analyzed for groups of animals, see Figure 3.

The pattern of imaging agent migration was initially similar between the treated and control animals, but rapidly became different (Fig. 2). As the experiment progressed week by week, the amount of migration to the spinal cord dramatically reduced in the oxaliplatin-treated animals relative to the controls.

**Figure 3:**
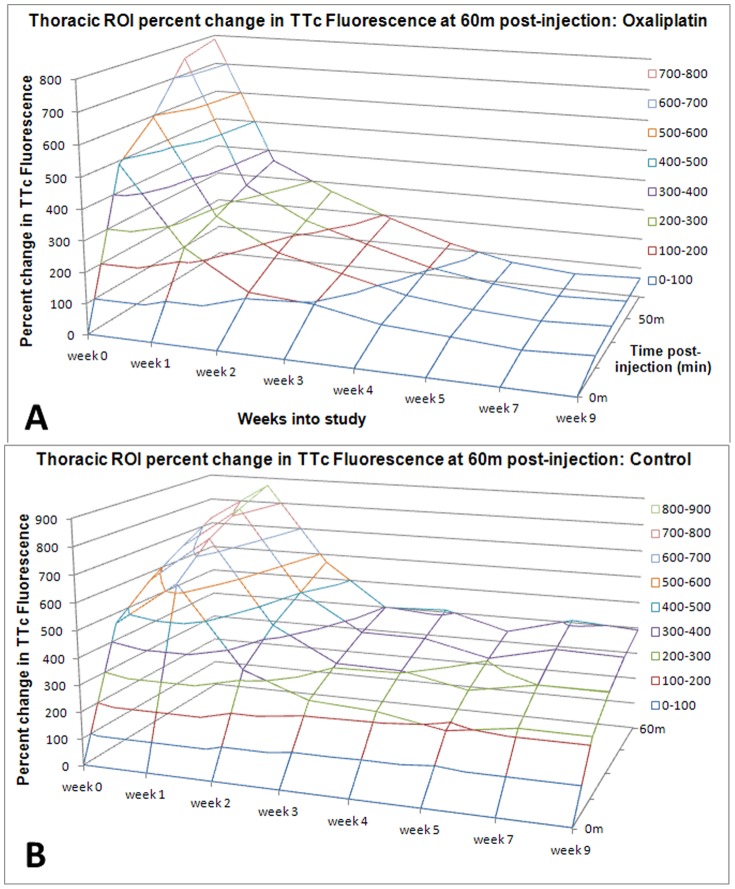
Oxaliplatin dramatically decreases the amount of nerve transport that occurs to the thoracic spine. This top graph illustrates time-fluorescent uptake curves as percent change from baseline (t = 0 min) through the course of the study for oxaliplatin treated animals (n = 8/group) (**A**). The same curves for sham-treated animals in **B** where the axes are scaled identically show the decrease in TTc transport starting at 1 week, and worsening steeply from there to the end of the study. Control animals also show a decrease of uptake from about 2 weeks onward, this is likely an experimental artifact related to repeated injections to the same body part.

Quantitative analysis of Regions of Interest (ROIs) placed over the spinal cord confirmed these findings, as demonstrated by wireplots of the uptake curves (Fig. 3), and in tabular form (Table 1), both for normalized data. Specifically, at week 0 there was an 8-fold increase in fluorescent activity over the thoracic spine from the baseline (t = 0 min) measurements to 60 minutes after TTc-790 injection in the calf, with no difference between treated and control groups (Fig. 2 for qualitative imaging result, Fig. 3 and Table 1 for quantitation of normalized data).

**Table 1:** Relative changes in Fluorescence.

Oxaliplatin	Time point (min)				
Weeks	0	30	40	50	60
**0**	0	489+/127%	595+/131%	754+/−146%	787+/−140%
**1**	0	236+/−67%	278+/−80%	328+/−88%	363+/−88%
**2**	0	112+/−36%	175+/−61%	223+/−81%	269+/−96%
**3**	0	97+/−32%	127+/−41%	161+/−50%	191+/−58%
**4**	0	57+/−18%	78+/−27%	103+/−34%	121+/−39%
**5**	0	44+/−15%	55+/−16%	69+/−21%	75+/−21%
**7**	0	29+/−7%	43+/−9%	51+/−14%	57+/−15%
**9**	0	39+/−12%	52+/−13%	58+/−14%	65+/−15%
**Control**	**Time point (min)**				
**Weeks**	**0**	**30**	**40**	**50**	**60**
**0**	0	462+/−79%	570+/−92%	650+/−106%	722+/−117%
**1**	0	631+/−126%	747+/−141%	817+/−149%	870+/−152%
**2**	0	332+/−79%	433+/−97%	502+/−106%	569+/−115%
**3**	0	243+/−66%	308+/−78%	361+/−86%	399+/−87%
**4**	0	229+/−70%	304+/−90%	359+/−100%	409+/−110%
**5**	0	186+/−58%	255+/−77%	304+/−89%	343+/−95%
**7**	0	223+/−64%	300+/−78%	362+/−87%	409+/−88%
**9**	0	222+/−73%	299+/−91%	355+/−102%	392+/−102%

Oxaliplatin dramatically decreases the amount of nerve transport that occurs to the thoracic spine. Observed mean percentage change (from t = 0) in fluorescence measured by ROI over the thoracic spine. Weeks indicate the time in weeks from the start of the experiment, time in minutes indicates the fluorescence measured at that time point (post-TTc injection) on the week of the experiment. This is the same data as shown in **Figures 3A and 3B**, but in tabular form (mean +/− standard deviation).

In oxaliplatin treated animals, however, there is a rapid loss of uptake from the injection site to the cord, starting from our first imaging time point at 1 week (corresponding to 5 doses of oxaliplatin, or a cumulative dose of 15 mg/kg) with fluorescence at 60 minutes has decreased to 4-fold the baseline value. The uptake continues to fall, reaching 2-fold by 3 weeks into the study and stays between 1.5–2 fold for the rest of the study out to 9 weeks (Fig. 3, Table 1).

**Figure 4:**
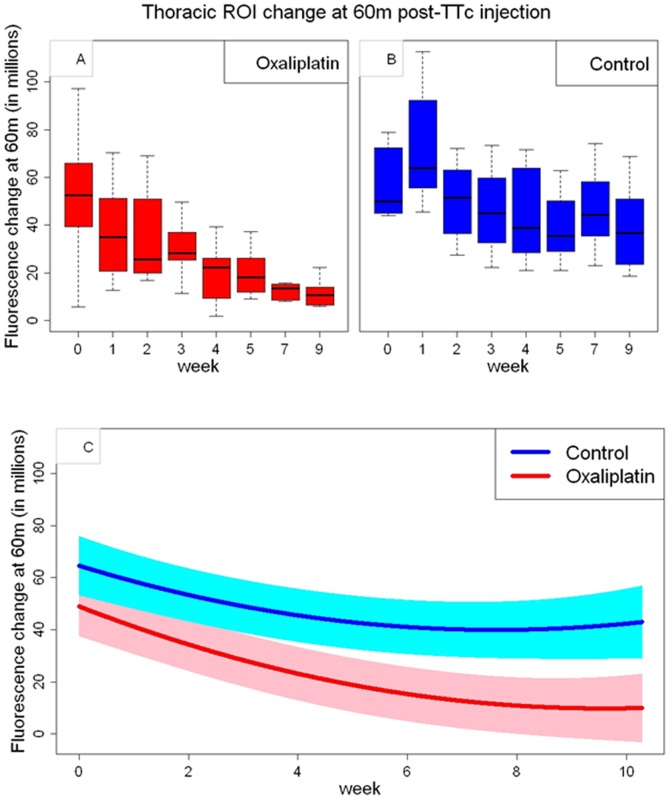
Observed and expected TTc uptake over time (replication week) for both oxaliplatin-treated and control animals demonstrates reduced transport for oxaliplatin-treated animals. The boxplots of the observed changes over time in fluorescent signal intensity in millions of photons per second per centimeter square per steradian (p s^−1^ cm^−2^ sr^−1^) with the ROI value at 0 minutes subtracted from the value at 60 minutes post TTc injection for oxaliplatin-treated to correct for background. Oxaliplatin (**A**) and control (**B**) animals are compared. Notice the relative maintenance uptake in control animals compared to a falling trend in the oxaliplatin treated animals, suggesting impaired transport in the latter. In results derived from the regression analysis (**C**) the solid lines represent the estimated mean change in fluorescent signal intensity for the two treatment groups, respectively. The shaded regions represent 95% pointwise confidence intervals. Notice how the 95% confidence intervals fail to overlap beyond 3 weeks, the point at which separation between the two groups becomes statistically significant.

Control animals also start out at an 8-fold increase of fluorescence over the thoracic spine at 60 minutes after injection. After a brief increase to 8.8-fold at 1 week, the values for control animals gradually decrease to slightly above 4-fold for the rest of the study (Fig. 3, Table 1). The uptake curves for oxaliplatin-treated and control animals are very different, as can be seen from a comparison of the uptake curves in the wiregraphs in Fig. 3A (oxaliplatin) and 3B (controls). Table 1 contains the relative change values plotted in Fig. 3. Raw absolute fluorescence values (not normalized) are plotted in Fig. 4, and tabulated in Table 2.

**Table 2:** Absolute Fluorescence measurements.

Oxaliplatin	Time point							
Weeks	0		30	40	50	60	p-value vswk 0 raw	p-value vswk 0 corrected
**0**	7.1+/−0.7		38.8+/−7.7	45.4+/−7.4	57.8+/−10.3	59.4+/−9.4	**n/a**	**n/a**
**1**	12.5+/−1.7		35.7+/−4.2	39.7+/−4.9	46.0+/−6.6	49.6+/−6.2	**0.404**	**0.221**
**2**	23.6+/−5.5		40.7+/−5.5	47.9+/−5.0	52.4+/−4.7	58.5+/−5.3	**0.932**	**0.164**
**3**	26.7+/−6.7		42.9+/−7.0	45.9+/−5.0	51.7+/−5.4	56.9+/−5.5	**0.821**	**0.059**
**4**	31.8+/−8.8		41.9+/−7.9	45.2+/−7.3	48.9+/−6.1	51.4+/−5.1	**0.468**	**0.011**
**5**	41.8+/−10.5		53.1+/−9.6	56.4+/−9.7	59.6+/−9.0	61.6+/−9.3	**0.872**	**0.011**
**7**	28.3+/−4.2		35.1+/−4.4	38.3+/−4.3	39.3+/−3.7	40.7+/−3.8	**0.097**	**0.004**
**9**	18.9+/−1.2		25.8+/−1.9	28.0+/−1.8	29.0+/−1.6	30.2+/−1.6	**0.017**	**0.003**
**Control**	**Time point**							
**Weeks**	**0**	**30**		**40**	**50**	**60**	**p-value** **vs wk 0** **raw**	**p-value** **vs wk 0** **corrected**
**0**	8.5+/−0.6	45.1+/−3.7		53.6+/−3.9	60.0+/−4.6	65.7+/−4.8	**n/a**	**n/a**
**1**	9.3+/−1.0	61.3+/−7.5		71.3+/−8.1	77.2+/−8.3	82.1+/−8.3	**0.116**	**0.153**
**2**	10.8+/−1.6	39.3+/−3.5		48.3+/−4.3	54.5+/−4.5	61.1+/−4.3	**0.485**	**0.385**
**3**	14.5+/−2.2	41.8+/−4.4		49.5+/−5.1	55.8+/−5.2	60.8+/−4.9	**0.484**	**0.198**
**4**	13.9+/−1.8	38.1+/−3.8		46.3+/−4.7	52.6+/−5.2	58.2+/−5.6	**0.329**	**0.166**
**5**	14.4+/−1.7	35.0+/−2.6		42.8+/−3.5	48.6+/−4.1	53.5+/−4.1	**0.076**	**0.031**
**7**	12.6+/−1.0	37.5+/−4.3		46.2+/−5.0	53.5+/−5.2	59.2+/−5.3	**0.379**	**0.200**
**9**	11.4+/−1.0	32.8+/−4.2		40.4+/−5.3	46.0+/−5.7	50.2+/−5.6	**0.054**	**0.043**

Observed mean fluorescence (in millions of photons per second per centimeter square per steradian (p s^−1^ cm^−2^ sr^−1^)) measured by ROI over the spine (A). Weeks indicate the time in weeks from the start of the experiment, time in minutes indicates the fluorescence measured at that time point (post-TTc injection) on the week of the experiment. P-values are for simple T-tests comparing values at 60 minutes between week 0 and every following week. Both raw values (absolute fluorescence at 60 minutes) and corrected values (fluorescence at 60 min minus fluorescence at 0 min) are compared.

Statistical analysis is given with simple T-tests in the rightmost columns of Table 2, where comparisons are made between the fluorescent measurements at 60 minutes for week 0 versus successive weeks. For oxaliplatin treated animals, background corrected fluorescence becomes statistically significantly different from week 0 at week 4 after treatment (p = 0.011) and thereafter. For controls, there are only two values statistically different from week 0 (those of weeks 5 and 9), without the clear trend seen for oxaliplatin treated animals. Comparing all values with T-tests in a matrix for uncorrected and background subtracted values yields Tables 3 and 4 respectively, in which the p-values can be seen to progressively get smaller as the weeks progress, in keeping with growing divergence between transport in oxaliplatin treated animals and controls.

**Table 3:** Comparing Absolute Fluorescence values for Oxaliplatin versus Controls.

p-values	Time point (min)				
Weeks	0	30	40	50	60
0	0.133	0.480	0.347	0.853	0.564
1	0.136	0.013	0.006	0.011	0.008
2	0.055	0.839	0.953	0.750	0.712
3	0.122	0.901	0.622	0.596	0.604
4	0.082	0.674	0.900	0.657	0.380
5	0.036	0.108	0.221	0.293	0.449
7	0.007	0.700	0.253	0.046	0.014
9	0.000	0.166	0.057	0.020	0.009

Raw, uncorrected values from Table 2 are used in this simple T-test comparison. Notice how p-values decrease from non-significant differences to significant differences as the number of weeks increase, corresponding to the growing divergence between treated and control animals.

**Table 4:** Comparing Corrected Absolute Fluorescence values for Oxaliplatin vs Controls.

p-values	Time point (min)				
Weeks	0	30	40	50	60
0	n/a	0.596	0.453	0.951	0.661
1	n/a	0.010	0.006	0.009	0.007
2	n/a	0.055	0.085	0.102	0.114
3	n/a	0.109	0.044	0.046	0.047
4	n/a	0.026	0.016	0.015	0.011
5	n/a	0.081	0.031	0.030	0.011
7	n/a	0.006	0.003	0.001	0.001
9	n/a	0.022	0.013	0.006	0.003

Raw values from Table 2 were corrected by subtracting the baseline fluorescence at time = 0 minutes in each experiment. Simple T-tests were run and p-values are shown below. Notice that initially there is no significant differences at week 0, but that p-values become progressively smaller as week number increases, and the treated and control groups diverge.

Analysis by means of a linear mixed statistical model (Fig. 4) confirms that transport is significantly impaired by oxaliplatin treatment compared to controls (p = 0.044), and this effect increases over time, as shown by a significant oxaliplatin-by-week interaction (p = 0.024) (note the different trends over time in the boxplots between Fig. 4A and B, the oxaliplatin and controls respectively). Divergence reaches statistical significance (p<0.05) at about 3 weeks into the study and thereafter (Fig. 4C, where the control and oxaliplatin groups no longer overlap).

An interesting aside was the observation of a significant trend (p = 0.013) to increase in fluorescence values at 0 minutes in oxaliplatin treated animals (Table 2, first column) as the study progresses, suggesting that some of the imaging agent does not clear out between imaging sessions, but persists in the animals to elevate the baseline. Table 2 shows elevated baseline fluorescence after 6 consecutive weeks of injections, peaking at week 5.

The uptake curves in controls animals did not stay stable over time either, though these changed much less than in the oxaliplatin treated animals. It appears that repeated injection of TTc into a single injection site reduces TTc transport as a function of time, even in controls. This effect holds for both the treatment and control groups (p<0.0001) and can be seen in the decreasing slope of values at 60 minutes for both the oxaliplatin and control groups in the wireplot (Fig. 3, and Fig. 4), with oxaliplatin falling more rapidly than controls.

The summary statistics for the fixed regression coefficient parameters of interest (in millions) are: intercept 64.4+/−5.22 (estimate +/− standard error), week −6.47+/−1.24, week squared 0.42+/−0.12, oxaliplatin −15.66+/−7.07 and oxaliplatin times week −1.69+/−0.74.

### Histology

**Figure 5:**
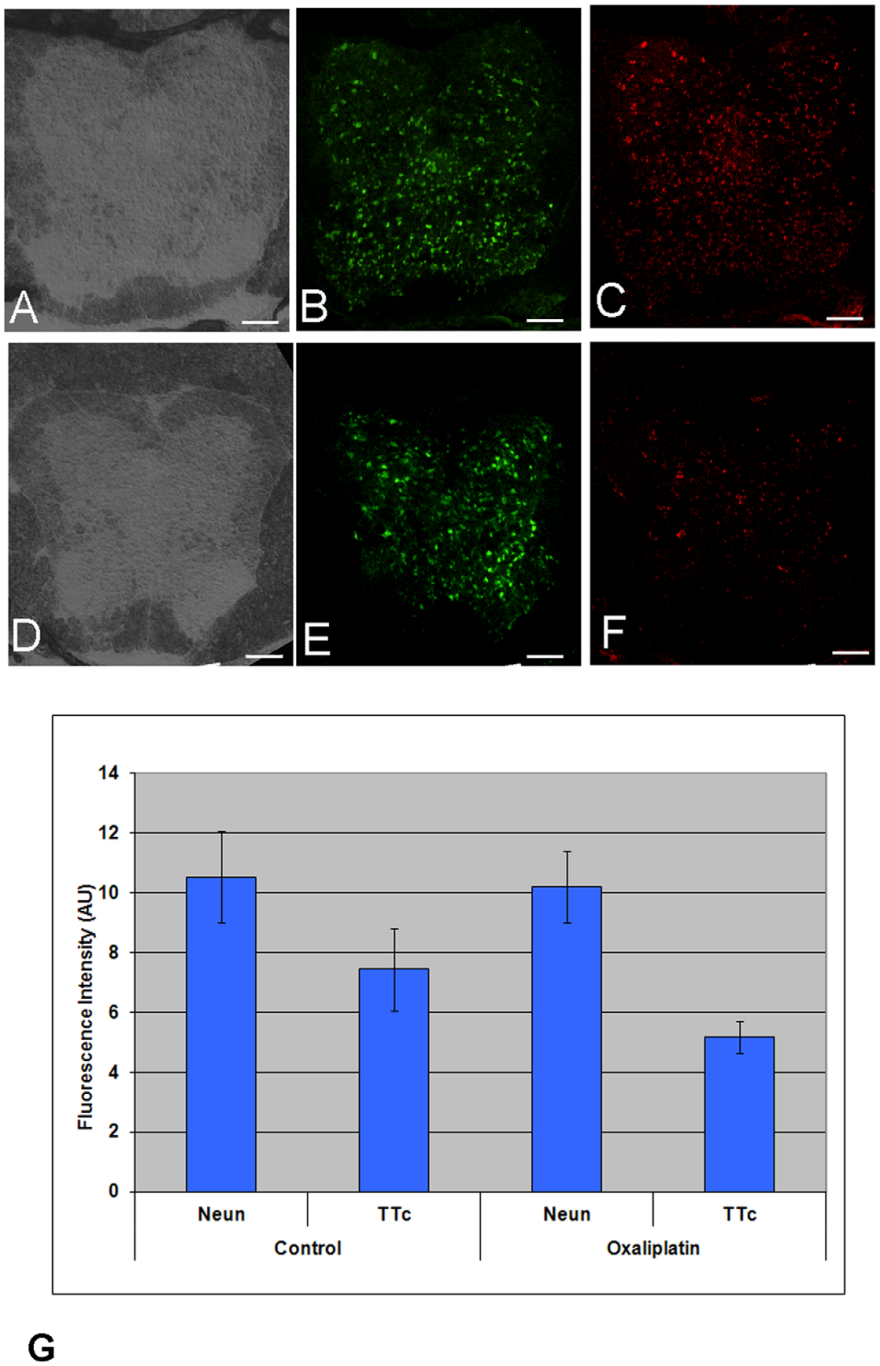
Oxaliplatin treatment reduces TTc transport, but not neuronal numbers. Immuno-histochemistry of representative spinal cord sections in normal (**A–C**) and oxaliplatin treated (**D–F**) animals at the S1 level. Cryosection block photographs (**A&D**), anti-NeuN immunofluorescence (B&E) and anti-TTc immunofluorescence (**C&F**) are compared and contrasted. The immuno-fluorescent anti-NeuN green fluorescence shows no difference between the control (**B**) and oxaliplatin (**E**) treated animals, indicating that oxaliplatin did not affect the number of neurons in the cord. However, the anti-TTc red fluorescence in non-treated animals (**C**) is much more prominent in comparison with the oxaliplatin treated animals (**F**), suggesting that TTc uptake and transport was affected by oxaliplatin treatment. Scale Bar = 100 µm Quantitative analysis of the histological data (**G**) (see text) shows no statistical difference for NeuN between control and oxaliplatin treated animals (p>0.56), but a significant decrease in TTc staining for oxaliplatin treated animals (p<0.0004).

Qualitative analysis of histology sections ranging from the 12^th^ thoracical (T12) to the 3^rd^ sacral-S3 vertebrae illustrate similar histological findings (Fig. 5). When control animal sections (Fig. 5A–C) and oxaliplatin treated animals (Fig. 5D–F) are compared, there is little difference in the level of NeuN staining a marker of neuronal cell mass (Fig. 5B&E), implying that at this point in our experiment there was little cord cell loss/death between groups. However, there was a significant reduction in TTc immuno-staining in oxaliplatin treated animals (Fig. 5F) in comparison with robust TTc-related immuno-staining in normal animals (Fig. 5C).

Quantitative analysis in Fig. 5G showed that for TTc, the mean fluorescence intensity after thresholding was 5.2 AU (+/−0.52) for the treatment group and 7.1 AU (+/−1.38) in the control group, a statistically significant difference (p<0.0004). For NeuN, the values were 10.2 AU (+/−1.21) for the treatment group and 10.5 AU (+/−1.53) for the control group, with no significant difference (p>0. 56). This indicates that the difference in TTc staining was not due to a difference in neuronal cell numbers (for example due to apoptosis/cell death), but rather due to differences in TTc uptake and transport.

## Discussion

We have previously shown that TTc can be used as neurographic imaging agent [27]. In this study we demonstrate for the first time -to our knowledge- the neurotoxic effects of oxaliplatin on neural transport by means of *in vivo* molecular imaging. We show that: a) neurotoxicity impairs neuronal transport, with statistically significant divergence occurring between treated animals and controls by 3 weeks, and b) that retrograde transport impairment appears to be irreversible up to the 9 weeks duration of our study. Our findings are not due to neuronal cell death, as NeuN staining of the cords showed equivalent neuronal cell mass in both groups, with reduced transport of TTc only noted in the Oxaliplatin treated group.

Tetanus toxin has been inferred, and its effects described from the times of the ancient Egyptians, but it was first isolated in 1967 [28]. While long suspected, the property of retrograde transport along the nerve was subsequently proven for tetanus toxin and its derivative peptides [29], [30], and eventually for the Tetanus Toxin C-fragment (TTc) [31], which seems to be the smallest subunit of the intact toxin that still has this property (an as an added benefit, TTc is non-toxic). Much work has been done to show how that TTc binds to sialic acid decorated gangliosides on neuronal membranes [32], [33], is transported by means of the fast axonal transport mechanism [31], [34], in a specialized vesicular compartment [35]. It was established that TTc is not specific for any specific type of nerve, but would be transported by all types, including motor, sensor and autonomic [36].

All platinum based chemotherapeutics can induce sensory neuropathy in cancer patients, with oxaliplatin being especially well known for cold induced allodynia [37], [38]. The literature has shown that human therapeutic doses can be achieved in rodents [20] and in cell culture [15]. We modeled our study after the dosage regimen established by Ta, et al [20], for which excellent functional and behavioral correlates are already known from the literature.

Imaging findings diverge between oxaliplatin treated and control animals almost from the beginning (Figs 2 and 3) with these differences reaching statistical significance after 3 weeks (area of non-overlap between the curves on Fig. 4, linear mixed-effects regression statistical model). This early divergence suggests that oxaliplatin’s disruption of neuronal transport occurs fairly early in the course of the neuropathy. Early oxaliplatin treatment effects are in agreement with findings in behavioral animal experiments using an identical drug regimen [20], showing the earliest neuropathic findings on functional cold plate testing to appear around one week and persist out to 6 weeks. Interestingly though, clinical findings (as reported in the literature) and imaging findings (as observed by us) appear to diverge later in the study as cold hyperalgesia was reported to resolve at about 6 weeks [20], but imaging showed a persistent decrease in neuronal transport out to the end of our 9 week study (Fig. 3).

Future work will be directed at investigating the very early events in neural transport, and correlating these to behavioral outcomes.

The phenomenon of subjective clinical improvement in the face of persistent neuropathy by objective measures (such as electromyographic studies) is well known, and likely reflects neuronal adaptation mechanisms to compensate for a loss of function. Such compensation can occur at brain [39], [40], [41], [42], spinal cord [43], or peripheral nerve [44] levels. Such adaptations can compensate up to a point, but beyond a threshold of neuronal loss, adaptations are no longer effective and clinical neurological deficits become apparent and fixed. Imaging findings will likely have important applications in defining the neuronal reserve capacity.

TTc uptake and transport of control animals did show some changes over time also, but to a much lesser degree than observed in oxaliplatin treated animals (Fig. 3). It is possible that the repeated injection of the imaging agent into a single localized area in the calf muscles over many weeks may cause local inflammation related to injection trauma, and this might interfere with the uptake of TTc in the later weeks of the experiment. This highlights the need for controls so that such effects can be discerned as separate from experimental variable effects (Fig. 4).

Elevations in baseline fluorescence (at 0 minutes, prior to injection) were observed over the course of the study in oxaliplatin treated animals (Table 2, first column), with a slow but significant increase in baseline fluorescence occurring to a peak at week 5 (p = 0.013). This increase as a function of time suggests that the oxaliplatin-treated animals had less clearance of TTc-related fluorescence than control animals, and might indicated that oxaliplatin interferes with the metabolism and excretion of TTc. The values at weeks 7 and 9 return closer to normal.

The linear mixed-effects regression analysis of fluorescence accounts for the baseline discrepancy over time by subtracting the measured value at 0 minutes.

Histological findings were consistent with imaging results, showing greatly reduced spinal cord TTc immunostaining in oxaliplatin treated animals compared to normal controls (Fig. 5). This independently confirms that TTc uptake and transport is impaired in oxaliplatin treated animals. Some residual staining remains present in treated animals, suggesting that the transport mechanism in oxaliplatin-treated animals is not completely shut down, but still present at some low level of activity. It is interesting to observe that both motor and sensory neurons showed reductions in TTc, indicating that the transport deficiency was not isolated to a single class of neurons. This is at variance with clinical observations showing sensory neuropathy only, with no motor component. Several interpretations of this data are possible: one, there might be a subclinical component of oxaliplating-induced neuropathy; two, transport deficits such as we observe are actually an epiphenomenon that has no relationship to neuropathy; and three, the relationships between transport defects and clinical syndromes are more complex than we currently appreciate. However, prior literature teaches that the transport of Nerve Growth Factor and TTc are transported in essentially the same way [45], with NGF restricted to autonomic or sensory nerves, but with TTc avidly transported in all nerves. This makes it likely that transport defects and nerve growth factor supply are related, but the relationship to neuropathy may be complex.

The mechanism of the neurotoxicity of oxaliplatin and other platinum based chemotherapeutics is unknown at present, but our work raises the hypothesis that impairment of the retrograde neuronal transport mechanism may be a primary event. This would favor the growth factor deficiency theory of neuropathic pathogenesis, and would suggest growth factor therapy (with or without a means to overcome neuronal transport difficulties) to treat or prevent neuropathy. Indeed, prior work has shown some attenuation of neuropathic effects by the administration of nerve growth factors [46], [47].

However this linkage may be complex, and other hypotheses, such as selective mitochondrial dysfunction in certain nerve types should also be considered [13], [14].

Primary abnormalities of axonal transport may be the underlying mechanism of oxaliplatin-induced neuropathy (and possibly other neuropathies). Molecular imaging offers to make this a testable hypothesis, and offers a measurable imaging parameter that can be targeted by interventions. The success of these interventions can be measured early in the course of the neuropathy and correlated to outcomes. These are the elements needed to develop a cure or preventative strategy for a disease in dire need of such interventions.

Our current fluorescent imaging technique could be used today as an intraoperative technique to mark and avoid nerves at risk for injury (assuming toxicological studies and regulatory approval to be in place), but limited photon penetration through tissue makes applications beyond the intra-operative setting difficult with fluorescent imaging. Other imaging modalities will be needed to take this technique beyond the operating room. With additional imaging modalities such as MR, however, neurographic molecular imaging could have wide general applicability as there are many currently incurable nerve conditions (diabetic neuropathy, amyotrophic lateral sclerosis, congenital neuropathies and many others) that could benefit from improved diagnosis and assessment of retrograde neuronal transport by means of imaging. It should be noted that recent major developments have been made in using fluorescent imaging (as yet in non-neurographic applications) in humans in the intra-operative setting [48], [49], we believe these to be the first of many similar translation events. Other approaches, such as the use of Cerenkov luminescent imaging might also be of interest [50], [51].

In conclusion, we demonstrate the usefulness of an *in vivo* molecular imaging tracer in the diagnosis and assessment of oxaliplatin-induced neuropathy, a frequently encountered problem in cancer patients. Our work suggests that molecular imaging is able to provide interesting mechanistic insights into the pathophysiology of neuropathy, specifically suggesting that impaired retrograde axonal transport, perhaps of growth factors, is an important part of this disease.

## References

[1] Andre T, Boni C, Mounedji-Boudiaf L, Navarro M, Tabernero J, et al (2004) Oxaliplatin, fluorouracil, and leucovorin as adjuvant treatment for colon cancer. New England Journal of Medicine 350: 2343–2351. 15175436 10.1056/NEJMoa032709

[2] Kurniali PC, Luo LG, Weitberg AB (2010) Role of calcium/magnesium infusion in oxaliplatin-based chemotherapy for colorectal cancer patients. Oncology (Williston Park) 24: 289–292. 20394142

[3] Saif MW, Reardon J (2005) Management of oxaliplatin-induced peripheral neuropathy. Ther Clin Risk Manag 1: 249–258. 18360567 PMC1661634

[4] Park SB, Krishnan AV, Lin CS, Goldstein D, Friedlander M, et al (2008) Mechanisms underlying chemotherapy-induced neurotoxicity and the potential for neuroprotective strategies. Curr Med Chem 15: 3081–3094. 19075655 10.2174/092986708786848569

[5] Cersosimo RJ (2005) Oxaliplatin-associated neuropathy: A review. Annals of Pharmacotherapy 39: 128–135. 15590869 10.1345/aph.1E319

[6] Wu SN, Chen BS, Wu YH, Peng H, Chen LT (2009) The mechanism of the actions of oxaliplatin on ion currents and action potentials in differentiated NG108–15 neuronal cells. NeuroToxicology 30: 677–685. 19422847 10.1016/j.neuro.2009.04.010

[7] Argyriou AA, Polychronopoulos P, Iconomou G, Chroni E, Kalofonos HP (2008) A review on oxaliplatin-induced peripheral nerve damage. Cancer Treat Rev 34: 368–377. 18281158 10.1016/j.ctrv.2008.01.003

[8] Egashira N, Hirakawa S, Kawashiri T, Yano T, Ikesue H, et al (2010) Mexiletine reverses oxaliplatin-induced neuropathic pain in rats. J Pharmacol Sci 112: 473–476. 20308797 10.1254/jphs.10012sc

[9] Scuteri A, Galimberti A, Ravasi M, Pasini S, Donzelli E, et al (2010) NGF protects Dorsal Root Ganglion neurons from oxaliplatin by modulating JNK/Sapk and ERK1/2. Neurosci Lett 486: 141–145. 20850503 10.1016/j.neulet.2010.09.028

[10] Parker AR, Petluru PN, Wu M, Zhao M, Kochat H, et al (2010) BNP7787-mediated modulation of paclitaxel- and cisplatin-induced aberrant microtubule protein polymerization in vitro. Mol Cancer Ther 9: 2558–2567. 20807779 10.1158/1535-7163.MCT-10-0300

[11] Kawashiri T, Egashira N, Watanabe H, Ikegami Y, Hirakawa S, et al (2011) Prevention of oxaliplatin-induced mechanical allodynia and neurodegeneration by neurotropin in the rat model. Eur J Pain 15: 344–350. 20829082 10.1016/j.ejpain.2010.08.006

[12] Kottschade LA, Sloan JA, Mazurczak MA, Johnson DB, Murphy BP, et al.. (2010) The use of vitamin E for the prevention of chemotherapy-induced peripheral neuropathy: results of a randomized phase III clinical trial. Support Care Cancer: in press. 10.1007/s00520-010-1018-3 PMC3329941 20936417

[13] Zheng H, Xiao WH, Bennett GJ (2011) Functional deficits in peripheral nerve mitochondria in rats with paclitaxel- and oxaliplatin-evoked painful peripheral neuropathy. Experimental Neurology 232: 154–161. 21907196 10.1016/j.expneurol.2011.08.016 PMC3202047

[14] Xiao WH, Zheng H, Zheng FY, Nuydens R, Meert TF, et al (2011) Mitochondrial abnormality in sensory, but not motor, axons in paclitaxel-evoked painful peripheral neuropathy in the rat. Neuroscience 199: 461–469. 22037390 10.1016/j.neuroscience.2011.10.010 PMC3237950

[15] Ta LE, Espeset L, Podratz J, Windebank AJ (2006) Neurotoxicity of oxaliplatin and cisplatin for dorsal root ganglion neurons correlates with platinum-DNA binding. NeuroToxicology 27: 992–1002. 16797073 10.1016/j.neuro.2006.04.010

[16] Cavaletti G, Marzorati L, Bogliun G, Colombo N, Marzola M, et al (1992) Cisplatin-induced peripheral neurotoxicity is dependent on total-dose intensity and single-dose intensity. Cancer 69: 203–207. 1309303 10.1002/1097-0142(19920101)69:1<203::aid-cncr2820690133>3.0.co;2-1

[17] Cavaletti G, Petruccioli MG, Tredici G, Marmiroli P, Barajon I, et al (1991) Effects of repeated administration of low doses of cisplatin on the rat nervous system. International Journal of Tissue Reactions 13: 151–157. 1960015

[18] Cavaletti G, Tredici G, Marmiroli P, Petruccioli MG, Barajon I, et al (1992) Morphometric study of the sensory neuron and peripheral nerve changes induced by chronic cisplatin (DDP) administration in rats. Acta Neuropathologica 84: 364–371. 1441917 10.1007/BF00227662

[19] Cavaletti G, Tredici G, Petruccioli MG, Dondè E, Tredici P, et al (2001) Effects of different schedules of oxaliplatin treatment on the peripheral nervous system of the rat. European Journal of Cancer 37: 2457–2463. 11720843 10.1016/s0959-8049(01)00300-8

[20] Ta LE, Low PA, Windebank AJ (2009) Mice with cisplatin and oxaliplatin-induced painful neuropathy develop distinct early responses to thermal stimuli. Mol Pain 5: 9. 19245717 10.1186/1744-8069-5-9 PMC2655284

[21] Ramos CR, Abreu PA, Nascimento AL, Ho PL (2004) A high-copy T7 Escherichia coli expression vector for the production of recombinant proteins with a minimal N-terminal His-tagged fusion peptide. Braz J Med Biol Res 37: 1103–1109. 15273812 10.1590/s0100-879x2004000800001

[22] Eisel U, Jarausch W, Goretzki K, Henschen A, Engels J, et al (1986) Tetanus toxin: primary structure, expression in E. coli, and homology with botulinum toxins. EMBO J 5: 2495–2502. 3536478 10.1002/j.1460-2075.1986.tb04527.x PMC1167145

[23] Fairweather NF, Lyness VA (1986) The complete nucleotide sequence of tetanus toxin. Nucleic Acids Res 14: 7809–7812. 3774547 10.1093/nar/14.19.7809 PMC311802

[24] Fairweather NF, Lyness VA, Pickard DJ, Allen G, Thomson RO (1986) Cloning, nucleotide sequencing, and expression of tetanus toxin fragment C in Escherichia coli. Journal of bacteriology 165: 21–27. 3510187 10.1128/jb.165.1.21-27.1986 PMC214364

[25] Sinha K, Box M, Lalli G, Schiavo G, Schneider H, et al (2000) Analysis of mutants of tetanus toxin Hc fragment: ganglioside binding, cell binding and retrograde axonal transport properties. Mol Microbiol 37: 1041–1051. 10972823 10.1046/j.1365-2958.2000.02091.x

[26] Watson C, Paxinos G, Kayalioglu G, editors (2008) The Spinal Cord: A Christopher and Dana Reeve Foundation Text and Atlas. 1 ed: Academic Press. 408 p.

[27] Schellingerhout D, Le Roux LG, Bredow S, Gelovani JG (2009) Fluorescence imaging of fast retrograde axonal transport in living animals. Mol Imaging 8: 319–329. 20003890

[28] Raynaud M, Turpin A, Bizzini B (1967) Properties of purified tetanus toxin and toxoid. Progress in immunobiological standardization 3: 244–247. 4985511

[29] Bizzini B, Grob P, Glicksman MA, Akert K (1980) Use of the B-IIb tetanus toxin derived fragment as a specific neuropharmacological transport agent. Brain Research 193: 221–227. 6155177 10.1016/0006-8993(80)90959-2

[30] Bizzini B, Grob P, Akert K (1981) Papain-derived fragment IIc of tetanus toxin: its binding to isolated synaptic membranes and retrograde axonal transport. Brain Research 210: 291–299. 6164441 10.1016/0006-8993(81)90902-1

[31] Fishman PS, Carrigan DR (1987) Retrograde transneuronal transfer of the C-fragment of tetanus toxin. Brain Research 406: 275–279. 3567626 10.1016/0006-8993(87)90792-x

[32] Louch HA, Buczko ES, Woody MA, Venable RM, Vann WF (2002) Identification of a binding site for ganglioside on the receptor binding domain of tetanus toxin. Biochemistry 41: 13644–13652. 12427026 10.1021/bi020291j

[33] Jayaraman S, Eswaramoorthy S, Kumaran D, Swaminathan S (2005) Common binding site for disialyllactose and tri-peptide in C-fragment of tetanus neurotoxin. Proteins 61: 288–295. 16104015 10.1002/prot.20595

[34] Grafstein B (1999) Intracellular traffic in nerve cells. Brain research bulletin 50: 311. 10643412 10.1016/s0361-9230(99)00157-4

[35] Bohnert S, Schiavo G (2005) Tetanus toxin is transported in a novel neuronal compartment characterized by a specialized pH regulation. Journal of Biological Chemistry 280: 42336–42344. 16236708 10.1074/jbc.M506750200

[36] Bizzini B, Stoeckel K, Schwab M (1977) An antigenic polypeptide fragment isolated from tetanus toxin: chemical characterization, binding to gangliosides and retrograde axonal transport in various neuron systems. Journal of neurochemistry 28: 529–542. 67185 10.1111/j.1471-4159.1977.tb10423.x

[37] Binder A, Stengel M, Maag R, Wasner G, Schoch R, et al (2007) Pain in oxaliplatin-induced neuropathy–sensitisation in the peripheral and central nociceptive system. Eur J Cancer 43: 2658–2663. 17855072 10.1016/j.ejca.2007.07.030

[38] Nassini R, Gees M, Harrison S, De Siena G, Materazzi S, et al (2011) Oxaliplatin elicits mechanical and cold allodynia in rodents via TRPA1 receptor stimulation. Pain 152: 1621–1631. 21481532 10.1016/j.pain.2011.02.051

[39] Chen XY, Wolpaw JR (2002) Probable corticospinal tract control of spinal cord plasticity in the rat. J Neurophysiol 87: 645–652. 11826033 10.1152/jn.00391.2001

[40] Wolpaw JR, Carp JS (2006) Plasticity from muscle to brain. Prog Neurobiol 78: 233–263. 16647181 10.1016/j.pneurobio.2006.03.001

[41] Wolpaw JR, Chen XY (2006) The cerebellum in maintenance of a motor skill: a hierarchy of brain and spinal cord plasticity underlies H-reflex conditioning. Learn Mem 13: 208–215. 16585796 10.1101/lm.92706 PMC1409832

[42] Fawcett J (2009) Molecular control of brain plasticity and repair. Prog Brain Res 175: 501–509. 19660677 10.1016/S0079-6123(09)17534-9

[43] Konya D, Liao WL, Choi H, Yu D, Woodard MC, et al (2008) Functional recovery in T13-L1 hemisected rats resulting from peripheral nerve rerouting: role of central neuroplasticity. Regen Med 3: 309–327. 18462055 10.2217/17460751.3.3.309

[44] Navarro X, Vivo M, Valero-Cabre A (2007) Neural plasticity after peripheral nerve injury and regeneration. Prog Neurobiol 82: 163–201. 17643733 10.1016/j.pneurobio.2007.06.005

[45] Stockel K, Thoenen H (1975) Comparison between the retrograde axonal transport of nerve growth factor (NGF) and tetanus toxin (TT) in sensory, motor and adrenergic neurons. Experimental Brain Research Vol 23, sup. 10.1016/0006-8993(75)90604-6 52914

[46] Schmidt Y, Unger JW, Bartke I, Reiter R (1995) Effect of nerve growth factor on peptide neurons in dorsal root ganglia after taxol or cisplatin treatment and in diabetic (db/db) mice. Experimental Neurology 132: 16–23. 7536686 10.1016/0014-4886(95)90054-3

[47] Apfel SC, Arezzo JC, Lipson L, Kessler JA (1992) Nerve growth factor prevents experimental cisplatin neuropathy. Annals of Neurology 31: 76–80. 1543351 10.1002/ana.410310114

[48] Troyan SL, Kianzad V, Gibbs-Strauss SL, Gioux S, Matsui A, et al (2009) The FLARE intraoperative near-infrared fluorescence imaging system: a first-in-human clinical trial in breast cancer sentinel lymph node mapping. Ann Surg Oncol 16: 2943–2952. 19582506 10.1245/s10434-009-0594-2 PMC2772055

[49] van Dam GM, Themelis G, Crane LM, Harlaar NJ, Pleijhuis RG, et al (2011) Intraoperative tumor-specific fluorescence imaging in ovarian cancer by folate receptor-alpha targeting: first in-human results. Nat Med 17: 1215–1219. 10.1038/nm.2472 21926976

[50] Spinelli AE, Kuo C, Rice BW, Calandrino R, Marzola P, et al (2011) Multispectral Cerenkov luminescence tomography for small animal optical imaging. Opt Express 19: 12605–12618. 21716501 10.1364/OE.19.012605

[51] Xu Y, Chang E, Liu H, Jiang H, Gambhir SS, et al (2012) Proof-of-Concept Study of Monitoring Cancer Drug Therapy with Cerenkov Luminescence Imaging. J Nucl Med 53: 312–317. 22241909 10.2967/jnumed.111.094623 PMC4143153

